# Physical Demands during Official Competitions in Elite Handball: A Systematic Review

**DOI:** 10.3390/ijerph20043353

**Published:** 2023-02-14

**Authors:** Carlos García-Sánchez, Rafael Manuel Navarro, Claude Karcher, Alfonso de la Rubia

**Affiliations:** 1 Deporte y Entrenamiento Research Group, Departamento de Deportes, Facultad de Ciencias de la Actividad Física y del Deporte (INEF), Universidad Politécnica de Madrid, C/Martín Fierro 7, 28040 Madrid, Spain; 2 Department of Sports Sciences, European University of Madrid, 28670 Villaviciosa de Odón, Spain; 3 Oxidative Stress and Muscular Protection Laboratory (EA 3072), Faculty of Medicine, Mitochondria, University of Strasbourg, 67081 Strasbourg, France; 4 European Centre for Education, Research and Innovation in Exercise Physiology (CEERIPE), Faculty of Sport Sciences, University of Strasbourg, 67000 Strasbourg, France; 5 Centre de Ressources, d’Expertises et de Performances Sportives, CREPS de Strasbourg, 67200 Strasbourg, France

**Keywords:** handball, physical demands, external load, internal load, workload, tracking system, LPS, IMU

## Abstract

An understanding of physical demands during official competitions is essential to achieving the highest performance in handball. The aim of this systematic review was to summarise the available scientific evidence associated with physical demands during official competitions in elite handball according to playing positions, competition level and gender. Following the Preferred Reporting Items for Systematic Reviews and Meta-Analyses guidelines, 17 studies were selected after a systematic search and selection process of three digital databases: PubMed, Web of Science and Sport Discus. The quality of the selected studies was evaluated using the Strengthening the Reporting of Observational Studies in Epidemiology checklist; the average score was 18.47 points. The sample consisted of 1175 handball players, of whom 1042 were men (88.68%) and 133 were women (11.32%). The results show that an elite handball player covered on average 3664.4 ± 1121.6 m during a match. The average running pace was 84.8 ± 17.2 m∙min^−1^. The total distance covered was largely greater in national competitions (4506.7 ± 647.9 m) compared with international competitions (2190.3 ± 1950.5 m) (effect size (ES) = 1.2); however, the running pace did not present any significant difference between the international or national level (ES = 0.06). In regard to gender, the total distance covered was moderately greater in female competitions (4549.1 ± 758.6 m) compared with male competitions (3332.6 ± 1257.7 m) (ES = 0.9), and the running pace was largely greater in female competitions (110.5 ± 7.2 m∙min^−1^) compared with male competitions (78.4 ± 19.7 m∙min^−1^) (ES = 1.6). In relation to playing position, backs and wings covered a moderately greater total distance (ES = 0.7 and 0.6) and slightly more meters per minute (ES = 0.4 and 0.2) than pivots. Moreover, the technical activity profile differed between playing positions. Backs performed moderately more throws than pivots and wings (ES = 1.2 and 0.9), pivots exhibited largely more body contact than backs and wings, and wings performed moderately more fast breaks (6.7 ± 3.0) than backs (2.2 ± 2.3) (ES = 1.8). Therefore, this research study provides practical applications for handball coaches and strength and conditioning professionals with respect to designing and implementing more individualised training programmes to maximise performance and reduce injury risk.

## 1. Introduction

Handball is a professional and Olympic sport played by two teams of seven players (six field players and one goalkeeper) on a court of 40 × 20 m. The game is characterised by fast-paced offensive and defensive actions and frequent body contact, with the ultimate objective of scoring more goals than the opponent at the end of two 30 min periods [1,2]. Therefore, the players must be physically prepared to produce actions to beat their opponents and to maintain the game’s speed and intensity throughout a match [2,3]. During the games, the players must perform different general movements (walking, running, jumping and changing directions) and handball-specific actions (passing, catching, throwing and blocking); specifically, in elite handball, there are different playing positions (backs, pivots, wings and goalkeepers) with different functions within the team, which generates different movement patterns and physical demands for each position [1,2,3].

Coaches must optimise different factors to achieve the maximum performance (i.e., players’ technical, tactical, psychosocial and physical characteristics) [4]. Thus, knowledge of the physical demands is one of the most relevant aspects of the game [3]. Understanding the physical demands is essential in order to optimise physical training (e.g., strength, speed and endurance), minimising the appearance of fatigue and reducing injury risk [3]. Moreover, to individualise training, it is worth nothing that playing positions, gender and competition level influence physical demands [1,2,3,5].

The study of physical demands in handball historically has been carried out mainly via the analysis of the external and internal load [5,6,7]. The most used variable for measuring external load was total distance covered (TDC). On the other hand, heart rate (HR), blood lactate concentrations (BLC) or the rating of perceived exertion (RPE) were the variables most used for measuring the internal load [8]. In relation to the technology, researchers traditionally [5,6] have mostly used time–motion analysis (TMA) to measure external load. This method is based on a video recording of the players during the game with the aim of coding actions and analysing behaviours and performance. Some authors have indicated that is an effective method for quantifying the physical demands of team handball [5], and it presents some advantages compared to observation sheets (e.g., high data processing capacity, reliability, accuracy, completely non-intrusive and no disruptions of the game) [9]. However, this method is time-consuming, and the quality of the data is dependent on the person; thus, it is an objective method when determining different locomotion speeds [10,11,12]. Furthermore, time–motion systems do not have a high enough precision for detecting and encoding high-intensity actions starting from low velocities, such as a maximal acceleration from a stop position. Therefore, this system can underestimate the physical demands during competitions [13].

Over the past two decades, the global positioning system (GPS) has been developed to understand, with greater accuracy and precision, the physical demands of outdoor team sports (i.e., football or rugby) [14]. However, this method cannot be used for indoor sports because the GPS signal is blocked [15,16]. Recently, with the purpose of overcoming this inconvenience, different companies (e.g., Wimu^TM^, Catapult^TM^ and Kinexon^TM^) developed a local positioning system (LPS) with ultra-wideband technology (UWB) [17] to track and analyse indoor team sports (i.e., basketball, handball, futsal, netball or ice hockey) [16,18,19,20]. A recent review of the scientific literature [21] clearly indicated the benefit of LPS over other systems, such as the widely used video-based systems. Furthermore, the majority of commercially available LPS devices now contain an inertial measurement unit (IMU) (e.g., accelerometer, magnetometer and gyroscope) recorder with a good level of validity [16,22,23,24,25]. IMU technology can offer accelerometery data including accelerations (ACC), decelerations (DEC), jumps, impacts and changes of direction (COD), an aspect for which video-based systems are not technologically prepared [15,20]. Moreover, these devices usually calculate a new variable from three-dimensional accelerometery data. Manufacturers have called this variable PlayerLoad, and for the past few years, it has been one of the most frequently used variables for monitoring external load in team sports [20,21,22,23,24,25].

In recent years, some researchers used GPS or LPS devices to examine the physical demands of handball [7,26,27,28,29,30,31,32,33,34,35]. This technology has been used to quantify training sessions [7,26], simulated or friendly matches [27,28,29], amateur competitions [30] and beach handball [31,32,33,34,35]. In the case of training sessions, researchers have tried to determine the physical demands of different small-sided games (3 vs. 3 and 6 vs. 6) [7,25]. In regard to friendly matches, researchers have tried to learn about physical demands in situations similar to official competitions, but without the difficulties associated with official games (requesting permission from clubs and federations, installing and calibrating tracking systems, etc.).

As mentioned above, researchers have used different methods and technologies to analyse the physical demands of handball. Accordingly, the aims of the present study are as follows: (i) summarise the available scientific evidence associated with physical demands during official competitions in elite men and women’s handball and (ii) examine the differences among playing position, competition level and gender.

## 2. Materials and Methods

The present systematic review was designed to synthesise the available scientific evidence about the analysis of physical demands in handball. The stages of the review process and subsequent analysis of the original articles stayed within the guidelines set out in the Preferred Reporting Items for Systematic Reviews and Meta-Analysis (PRISMA) [36] checklist and the Population, Interventions, Comparisons, Outcomes and Study Design (PICOS) question model for the definition of inclusion criteria.

### 2.1. Study Selection and Eligibility Criteria

Primary and original studies that analysed physical demands in handball were included. Studies were published in any language and in peer-reviewed journals with an impact factor included in the Journal Citation Reports of the Web of Science until September 2022.

According to the ‘PICOS’ question model, the five inclusion criteria are as follows: (1) ‘Population’: male and female players belonging to the first competition level (top-tier professional leagues or tours—international level), second level (second-tier professional leagues or tours—national level) or third level (players involved in talent development processes) [37]; (2) ‘Intervention’: studies with information about physical demands in official competitions; (3) ‘Comparison’: differences among gender, playing positions and competition level; (4) ‘Outcomes’: internal or external load measurement or technical activity; (5) ‘Study Design’: a descriptive observational study.

The four exclusion criteria are as follows: (1) analysed training demands or game-based training demands; (2) analysed simulated or friendly matches; (3) investigated the physical demands in beach handball; (4) the sample was composed of amateur handball players or students. Systematic reviews, reviews, letters to the editor, articles that were not peer-reviewed, editorials, books, periodicals, surveys, opinion pieces and conference abstracts were considered as support materials in the search process for potentially valid research according to the aim of the study.

### 2.2. Literature Search

The selection process of scientific studies was conducted by searching three electronic databases: Web of Science, PubMed and Sport Discus. A systematic and computerised literature search was performed between July 2022 and September 2022 to register all relevant scientific articles investigating the physical demands of handball. The following Boolean search strategy was applied using operators ‘AND’ and ‘OR’: (“internal load” OR “external load” OR “workload” OR “physical demands” OR “activity profile” OR “match profile” OR “match analysis”) AND (“handball”). Moreover, studies were incorporated using additional sources (bibliography of systematic reviews and alerts received by e-mail during the process).

### 2.3. Systematic Review Protocol

**Figure 1:**
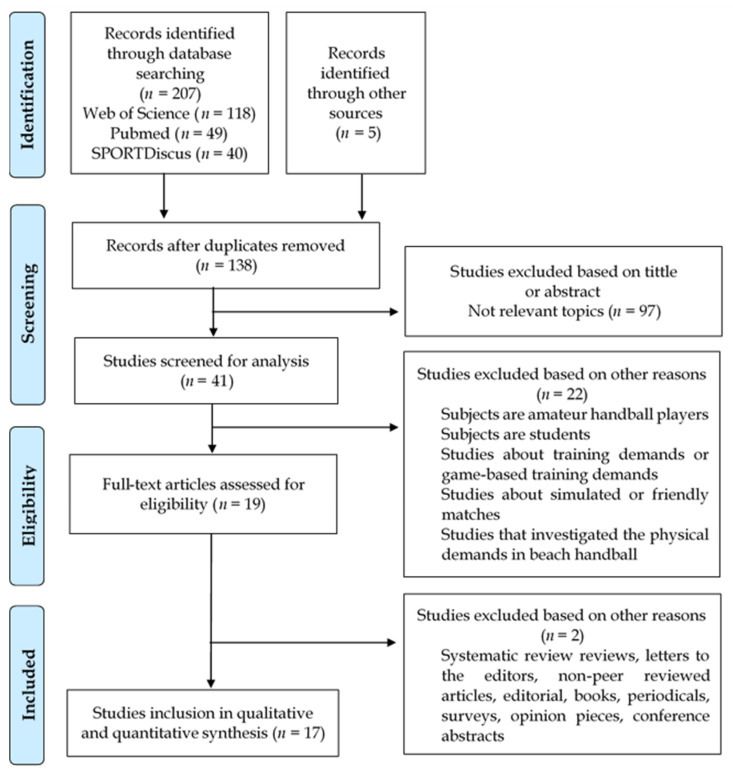
Flow diagram for screening and selection of studies according to Preferred Reporting Items for Systematic Reviews and Meta-Analysis (PRISMA).

The authors worked separately and independently to ensure the reliability of the process and the suitable eligibility of the studies. According to PRISMA criteria [36], the protocol was carried out in the months of July, August, and September 2022 and comprised four stages (Figure 1): (1) identification: the first author (C.G.S.) found 207 studies in the 3 digital databases—five additional articles were identified from the reference lists of included papers and review articles that were already published; (2) screening: the first author (C.G.S.) eliminated the duplicate files (*n* = 74) and excluded those considered not relevant via a previous review of the title, abstract and keywords (*n* = 97); (3) eligibility: the first (C.G.S.), second (A.d.l.R.) and third author (R.M.N.) eliminated full-text studies from the selection process by the eligibility criteria (*n* = 22); (4) inclusion: the remaining studies (*n* = 17) were finally considered suitable.

### 2.4. Data Extraction and Management

The information extracted from the studies included in this systematic review was distributed in the following categories and subcategories: (A) sample characteristics: (A1) number of players, (A2) playing positions (wing, pivot, back and goalkeeper), (A3) competitive level (international or first level, national or second level, talent development processes or third level), (A4) gender (male and female), (A5) age, (A6) body mass, (A7) height, (A8) body fat and (A9) experience; (B) methods: (B1) data records, and (B2) technology; (C) external load measured with TMA: (C1) standing still, (C2) walking, (C3) jogging, (C4) running, (C5) high-intensity running and (C6) sprinting; (D) external load measured with LPS: (D1) playing time, (D2) total distance covered, (D3) running pace, (D4) high-intensity running, (D5) sprinting, (D6) high-intensity accelerations, (D7) high-intensity decelerations, (D8) high-intensity events per minute, (D9) player load and (D10) player load per minute; (E) internal load: (E1) heart rate mean, (E2) heart rate peak and (E3) blood lactate concentration; (F) technical activity: (F1) jumps, (F2) throws, (F3) stops, (F4) changes of direction, (F5) one-on-one situations, (F6) fast breaks, (F7) tackles and (F8) screenings.

### 2.5. Study Quality Assessment

The quality of the studies was evaluated using the Strengthening the Reporting of Observational Studies in Epidemiology (“STROBE”) criteria [38]. This checklist is composed of 22 items clustered into 6 categories belonging to different sections of the study: ‘Title–Abstract’ (Item 1), ‘Introduction’ (Items 2–3), ‘Methods’ (Items 4–12), ‘Results’ (Items 13–17), ‘Discussion’ (Items 18–21) and ‘Funding’ (Item 22). A score of ‘0’ was assigned to incomplete items, and ‘1’ was assigned to items that were described accurately. The overall rating was obtained from the summation of the item values based on the following levels: ‘very low quality’ (0–4 points), ‘low quality’ (5–8 points), ‘medium quality’ (9–12 points), ‘high quality’ (13–16 points) and ‘very high quality’ (17–22 points). The quality assessment of the studies was carried out by two independent reviewers (C.G.S. and A.d.l.R.). Another reviewer (R.N.) resolved disagreements in the rating, and inter-rater reliability was calculated.

### 2.6. Statistical Analysis

Data analysis was performed using SPSS for Windows (Version 26, IBM Corp., Armonk, NY, USA). The values shown for the quantitative and ordinal variables are expressed as mean ± standard deviation (M ± SD) values. Standardised differences in match demands between playing positions, gender, and competition level (or effect sizes [ES] [39]) have been calculated when data were available, and they were interpreted using Hopkins’ categorisation criteria, where 0.2, 0.6, 1.2 and >2 are considered small, moderate, large and very large effects, respectively [39].

## 3. Results

### 3.1. Summary of Scientific Evidence (Qualitative Analysis)

Scientific evidence on the sample characteristics (A), methods (B), external load (C, D) and internal load (D) is shown in Table 1, Table 2, Table 3 and Table 4. The studies are shown in alphabetical order.

#### 3.1.1. Sample Characteristics and Methods

Table 1 shows the sample characteristics and methods. The 17 studies included in this systematic review comprised data of 1175 handball players, of whom 88.68% (*n* = 1042) were men and 11.32% (*n* = 133) were women. The mean age of the total sample was 25.54 ± 3.28. Regarding the competitive level, six studies were developed in international competitions (*n* = 901), ten studies were conducted in national leagues (*n* = 260) and one study was conducted in a youth elite competition (*n* = 14). Finally, nine studies reported the players’ competitive experience, and five studies no specified the playing positions. Only three studies reported information about body fat.

In relation to the data records, 269 HR samples and 50 BLC samples were included, providing information about the players’ internal load. On the other hand, 3244 TMA records in 342 matches and 2480 LPS records in 121 matches were collected with the aim of understanding the players´ external load. Regarding the technology used, ten studies (58.82%) were developed with TMA, and six studies (35.29%) were developed with LPS.

#### 3.1.2. External Load

Table 2 shows the external load data measured with LPS.

**Figure 2:**
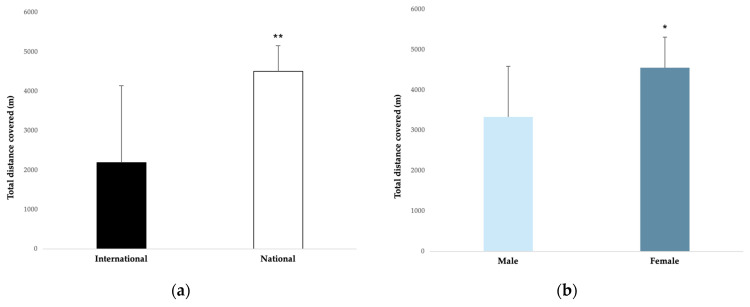
(**a**,**b**). Total distance covered according to the competition level and gender. The magnitude of the standardised differences (effect size) between the competition level and gender is indicated by the number of symbols: one symbol stands for a moderate difference and two for a large difference.

One of the main findings was that an elite handball player covered an average of 3664.4 ± 1121.6 m during a match. However, the results obtained for TDC were heterogeneous (range 1346.6 ± 3852.5 to 6943.9 ± 364.4 m). Moreover, in terms of competition level, TDC was largely greater in national competitions (4506.7 ± 647.9 m) compared with international competitions (2190.3 ± 1950.5 m) (ES = 1.2). In relation to the gender, TDC was moderately greater in female competitions (4549.1 ± 758.6 m) compared with male competitions (3332.6 ± 1257.7 m) (ES = 0.9) (Figure 2a,b).

**Figure 3:**
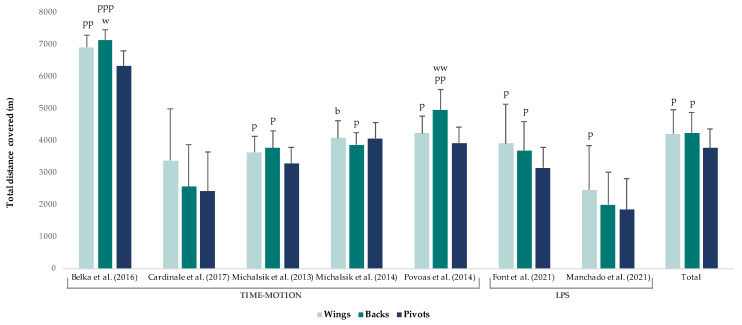
Total distance covered according to playing positions. The magnitude of the standardised differences (effect size) between the different positions is indicated by the number of symbols: one symbol stands for a moderate difference, two for a large difference and three for a very large difference. b stands for substantial difference vs. backs, w vs. wings and p vs. pivot [10,12,15,40,42,43,48].

TDC differed according to playing positions. Backs (ES = 0.7) and wings (ES = 0.6) covered a moderately greater total distance than pivots (Figure 3).

Regarding the locomotor categories, handball players spent more than 50% of total playing time walking or standing still; in contrast, they spent less than 10% of the total playing time running at high intensity and less than 5% for sprinting. Furthermore, in relation to gender, some studies [42,43] indicated that male players covered more distance at high velocities (high-intensity running and sprinting) than female players. By comparison, female players covered more distance at low velocities (walking and jogging) (Table 3).

**Figure 4:**
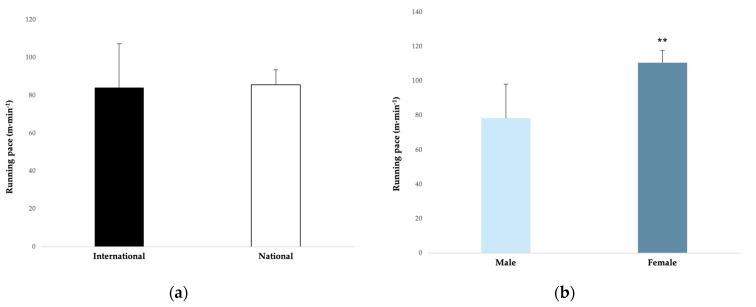
(**a**,**b**). Running pace related to the competition level and gender. The magnitude of the standardised differences (effect size) between the competition level and gender is indicated by the number of symbols: two for a large difference.

In relation to running pace, an elite handball player covered an average of 84.8 ± 17.2 m∙min^−1^ during a match. In terms of competition level, running pace did not present any significant difference between international and national levels (ES = 0.06). In relation to gender, the running pace was largely greater in female competitions (110.5 ± 7.2 m∙min^−1^) compared to male competitions (78.4 ± 19.7 m∙min^−1^) (ES = 1.6) (Figure 4a,b).

**Figure 5:**
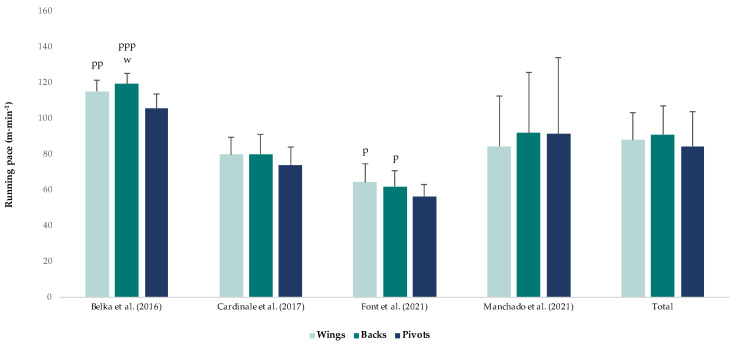
Running pace according to playing positions. The magnitude of the standardised differences (effect size) between the different positions is indicated by the number of symbols: one symbol stands for a moderate difference, two for a large difference, three for a very large difference. B stands for substantial difference vs. backs, w vs. wings and p vs. pivot [10,12,15,40].

Running pace presented small differences between playing positions. Backs (ES = 0.4) and wings (ES = 0.2) covered slightly more meters per minute than pivots (Figure 5).

Font et al. [15] found that handball players performed more than 1000 accelerations and decelerations during a game [15], and Luteberget et al. [13] found an average of 3.9 ± 1.5 high-intensity events per minute (sum of the ACC, DEC and COD greater than 2.5 m∙s^−2^). Wings, backs and pivots performed a similar number of accelerations and decelerations [15]. However, high-intensity accelerations (HIA) and high-intensity decelerations (HID) were different between playing positions [15]. Wings (134.8 ± 60.7) performed slightly more HIA than backs (128.1 ± 55.2) and pivots (112.0 ± 33.6) (ES = 0.12 and 0.46, respectively); in contrast, backs (114.6 ± 51.77) performed a similar number of HID than wings (112.9 ± 56.0) and slightly more than pivots (99.6 ± 28.9) (ES = 0.03 and 0.3, respectively).

In regard to contextual factors, some studies indicated differences in external load outcomes between the first and second half of the match; specifically, the time spent in high-intensity movements and in high-intensity running during the match decreased in the second half [42,43,47]. Moreover, the total distance covered in the first 10 min was slightly higher than what was covered in the last 10 min of the game (ES = 0.33) [10]. In addition, the initial values of PlayerLoad/min declined throughout halves [50].

Furthermore, some researchers indicated that there were no differences between top-ranked and lower-ranked teams [10,12] or between winners and losers [12] in the total distance covered and running pace during games.

#### 3.1.3. Internal Load

**Figure 6:**
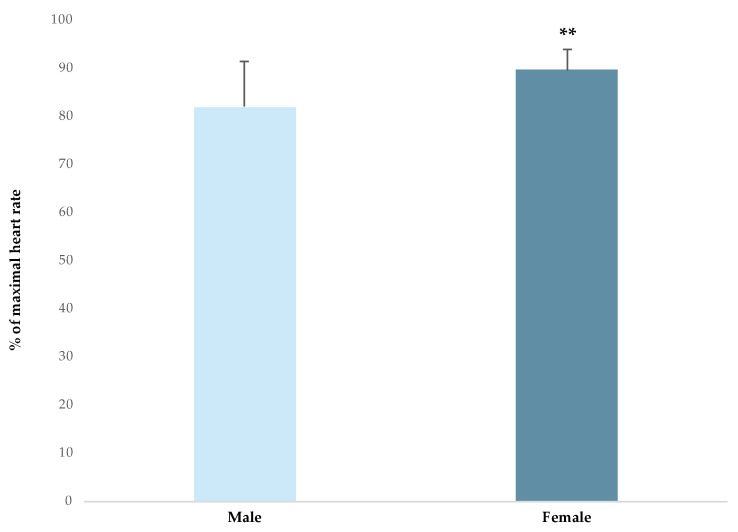
Heart rate responses according to the gender. The magnitude of the standardised differences (effect size) between the competition level and gender is indicated by the number of symbols: two for a large difference.

Table 4 shows the internal load outcomes (HR mean, HR peak and BLC). In the studies included in this systematic review, the average heart rate ranges from 70 ± 11.0% to 90.1 ± 4.3% of heart rate maximum (%HRmax). In regard to the playing position, Povoas et al. [48] found that backs and pivots have slightly higher HR mean values compared with wings (ES = 0.5 and 0.4, respectively). Moreover, backs, pivots and wings have moderately higher HR mean values compared with goalkeepers (ES = 1.3, 1.2, and 0.9, respectively). In the same line, Manchado et al. [9] found that field players (backs, pivots and wings) have a moderately higher HR mean compared with goalkeepers (ES = 1.8). In contrast, Belka et al. [40] found similar HR mean values in backs, pivots and wings. In regard to gender, the HR mean was moderately greater in female players (89.7 ± 4.1 %HRmax) compared with male players (82.0 ± 9.3 %HRmax) (ES = 1.0) (Figure 6).

The mean BLC ranges from 3.6 ± 2.1 to 4.8 ± 1.9 mmol·L^−1^; however, at some points in the match, the BLC exceeded 8.0 mmol·L^−1^ [44,49]. Furthermore, some studies indicated differences in the internal load responses between the first and second half of the match, specifically the percentage of time spent on exercise intensities >80% HRmax decreased during the second half [9,47,48,49].

#### 3.1.4. Technical Activity

**Figure 7:**
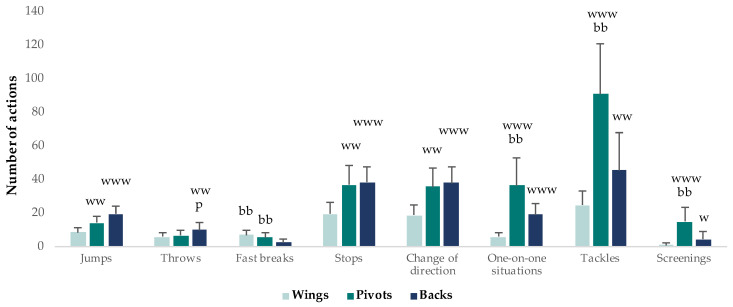
Technical activity according to playing positions. The magnitude of the standardised differences (effect size) between the different positions is indicated by the number of symbols: one symbol stands for a moderate difference, two for a large difference and three for a very large difference; b for substantial difference vs. backs, w vs. wings and p vs. pivot. Data were merged from different studies: throws data come from Michalsik et al. [45,46] and Povoas et al. [48]; fast breaks from Michalsik et al. [45,46]; jumps, stops, COD and one-on-one situations from Povoas et al. [48]; tackles and screenings from Michalsik et al. [46].

The technical activity profile differed among playing positions. Backs and pivots performed moderately more jumps, stops and COD during a game than wings. Moreover, backs performed moderately more throws (9.9 ± 4.1) than pivots and wings (6.6 ± 2.8 and 5.7 ± 2.4, respectively) (ES = 1.2 and 0.9, respectively). On the other hand, pivots exhibited largely more body contact (one-on-one situations, tackles and screenings) than backs and wings. Finally, wings and pivots performed moderately more fast breaks (6.7 ± 3.0 and 5.4 ± 2.9, respectively) than backs (2.2 ± 2.3) (ES = 1.8 and 1.2, respectively) (Figure 7).

In addition, the number of actions per match presented some differences and similarities between male and female players. Male players performed largely more fast breaks (6.9 ± 3.3) [46] than female players (5.2 ± 2.8) (ES = 1.3) [45], but they have a similar performance in throwing activity (7.0 ± 3.2 for male players vs. 7.7 ± 3.6 for female players) (ES = 0.2) [45,46,47,48].

### 3.2. Study Selection and Quality Assessment (Qualitative Analysis)

The quality analysis (‘STROBE’ checklist) yielded the following results (Table 5): (a) the quality scores ranged from 16 to 21; (b) the average score was 18.47 points; (c) of the 17 included studies, 3 (17.65%) were considered ‘high quality’ (13–16 points); fourteen (82.35%) were categorised as ‘very high quality’ (17–22 points); (d) the highest scores (14 points or 100%) were located on Items 1, 2, 3, 5, 6, 7, 8, 11, 12, 13, 15, 16, 18 and 20. In contrast, the most commonly absent or incomplete items were No. 9 (0 points, or 0%) and No. 17 (7 points, or 41.18%).

## 4. Discussion

To our knowledge, the present study represents the most comprehensive and exhaustive systematic review for analysing physical demands during official competitions in elite male and female handball. The main strengths of this research lie in the qualities of external validity and generalisability of the results from the analysis of a large sample of handball players (*n* = 1175), TMA records (*n* = 3244) and LPS register (*n* = 2480), as well as a strict evaluation of the published data. However, the final sample comprised a small number of female players (*n* = 133), and no studies were carried out at the Olympic Games. On the other hand, a reduced number of HR (*n* = 269) and BLC (*n* = 50) samples were collected.

Furthermore, from a methodological perspective, this systematic review presents a robust design based on the search process carried out in three databases and limited to unambiguous inclusion and exclusion criteria. Moreover, 14 of the 17 included studies were categorised as ‘very high quality’ (*n* = 14), and 3 were categorized as ‘high quality’ (*n* = 3) according to the STROBE checklist.

### 4.1. External Load

#### 4.1.1. Total Distance Covered

The total distance covered was monitored in 13 of the 17 studies included in this systematic review. This finding confirms that the total distance covered is, historically, one of the first variables to be monitored and the most commonly used variable for measuring external load in many sports [51,52,53,54]. Our results show that an elite handball player covered an average of 3664.4 ± 1121.6 m during a match (from 1346.6 ± 3852.5 to 6943.9 ± 364.4 m). We found similar values in terms of TDC in all studies included in this systematic review, except in the study of Belka et al. [40], where a higher distance covered was observed in all positions. We hypothesised that this result could be explained by two reasons: (i) the study was developed with time–motion analysis, which has lower accuracy than LPS [13,21]; (ii) the sample is made up of female young players and perhaps the game pace of their competition is higher because of technical fouls [55,56,57].

In terms of competition level, TDC was largely greater in national competitions (4506.7 ± 647.9 m) compared with international competitions (2190.3 ± 1950.5 m). These results may indicate a larger use of rotations and thus less playing time in national team players, because this type of competition has a high density of games in a relatively short period of time [10]. In regard to gender, TDC was moderately greater in female competitions (4549.1 ± 758.6 m) compared with male competitions (3332.6 ± 1257.7 m); as we mentioned above, perhaps the game pace of female competition is higher because of technical fouls [55,56,57]. Finally, according to playing positions, wings and backs covered a moderately greater distance than pivots, regardless of the technology used to measure it (TMA or LPS) [10,12,15,40,42,43,48].

#### 4.1.2. Running Pace

The running pace was measured only in 5 of the 17 studies included in this systematic review, although this variable is especially relevant in sports with multiple substitutions such as handball, because each player spends a different amount of time on the court. Thus, we should normalise the distance covered according to the time the players spend on the court to obtain a true reflection of real demands [10,11,50]. The results of this systematic review show that an elite handball player covered an average of 84.8 ± 17.2 m∙min^−1^ during a match. In regard to the phase of the game, Manchado et al. [11,12] indicated that the running pace is significantly higher in offense (88.45 ± 20.72 m∙min^−1^ and 96.53 ± 22.57 m∙min^−1^, respectively) than in defence (80.83 ± 27.11 m∙min^−1^ and 82.72 ± 43.28 m∙min^−1^, respectively) (ES = 0.47 and 0.32, respectively). This fact could indicate that specialist players (offense or defence) should have a different training programme and should focus on developing the ability to accelerate and to decelerate in short distances, to move in limited spaces and to bear heavy body contact [10].

In terms of competition level, the running pace did not present any significant difference between the international or national levels. However, competition level affects running pace in men’s competitions. International men’s competitions had a higher running pace (84.1 ± 23.3 m∙min^−1^) [10,11,12] than national men’s competitions (61.3 ± 9.1 m∙min^−1^) [15]. This difference could be explained by the greater intensity and speed of the game in international competitions. In regard to gender, the running pace was largely greater in female competitions (110.5 ± 7.2 m∙min^−1^) compared with male competitions (78.4 ± 19.7 m∙min^−1^). Finally, the running pace presented small differences between playing positions. For example, Font et al. [15] and Belka et al. [40] found more running pace for wings (64.5 ± 10.4 m∙min^−1^ and 115.3 ± 6.2 m∙min^−1^, respectively) and backs (61.9 ± 8.7 m∙min^−1^ and 119.4 ± 6.1 m∙min^−1^, respectively) compared with pivots (56.5 ± 6.6 m∙min^−1^ and 105.6 ± 8.1 m∙min^−1^, respectively). Likewise, Cardinale et al. [10] found more running pace for centre backs (82.1 ± 11.8 m∙min^−1^) and wings (79.7 ± 9.7 m∙min^−1^) compared with backs (77.9 ± 10.7 m∙min^−1^) and pivots (74.0 ± 9.8 m∙min^−1^). On the other hand, Manchado et al. [12] found more running pace for centre backs (98.34 ± 36.11 m∙min^−1^) and pivots (91.17 ± 42.67 m∙min^−1^) compared with backs (88.7 ± 32.8 m∙min^−1^) and wings (85.1 ± 32.9 m∙min^−1^).

#### 4.1.3. High-Intensity Running and Sprinting

Several studies [10,12,15,42,43,47,48] indicated that the time handball players spent walking or standing was still more than 50% of total playing time; in contrast, they spent less than 10% of total playing time running at high intensity or sprinting. Although these high-intensity actions (running or sprinting) represent a small percentage with regard to the total, they are generally crucial for game outcomes (e.g., sprinting to win a ball and sprinting during counterattacks) [3]. In addition, different studies demonstrating that the recovery time between high-intensity efforts ranges between 90 and 120 s [10,47,48]. These findings suggest that the work-to-rest ratio or the work-recovery ratio between high-intensity actions is enough for phosphocreatine (PCr) re-synthesis in handball. This fact would allow players to maintain the high-energy required to avoid a performance decrement in high-intensity actions throughout the game [3,58,59].

In regard to gender, Michalsik et al. [42,43] found that male players covered higher distances at high velocities (high-intensity running and sprinting) than female players, but female players covered more distance at low velocities (walking and jogging). This difference could be explained by the higher number of fast breaks performed by male players compared with female players [45,46]. Moreover, according to playing positions, wings covered largely greater distances at high-intensity running and sprinting than backs and pivots [10,11,12,15,40,42]; these results could be related to their increased participation in the counter-attack phase [45,46]. Finally, in regard to the phase of the game, Manchado et al. [12] reported a higher percentage of high-intensity actions in offense compared with defence. Therefore, strength and conditioning coaches should consider this information when designing optimal training programmes according to gender and playing positions.

#### 4.1.4. Acceleration, Deceleration and PlayerLoad

Data regarding accelerations and decelerations during the handball matches were scarce; specifically, in our systematic review, only two studies provided information about accelerometery. Font et al. [15] found that handball players performed more than 1000 accelerations and decelerations during a game [15], and Luteberget et al. [13] found an average of 3.9 ± 1.5 high-intensity events per minute (sum of the ACC, DEC and COD greater than 2.5 m∙s^−2^). Therefore, the ability to accelerate, decelerate and change direction properly is mandatory for successful performance in handball [60]. Accordingly, the ability to accelerate is more important for successful performances than maximum velocity, probably because handball players rarely had enough space to achieve maximum speed during competitions [15,61].

**Figure 8:**
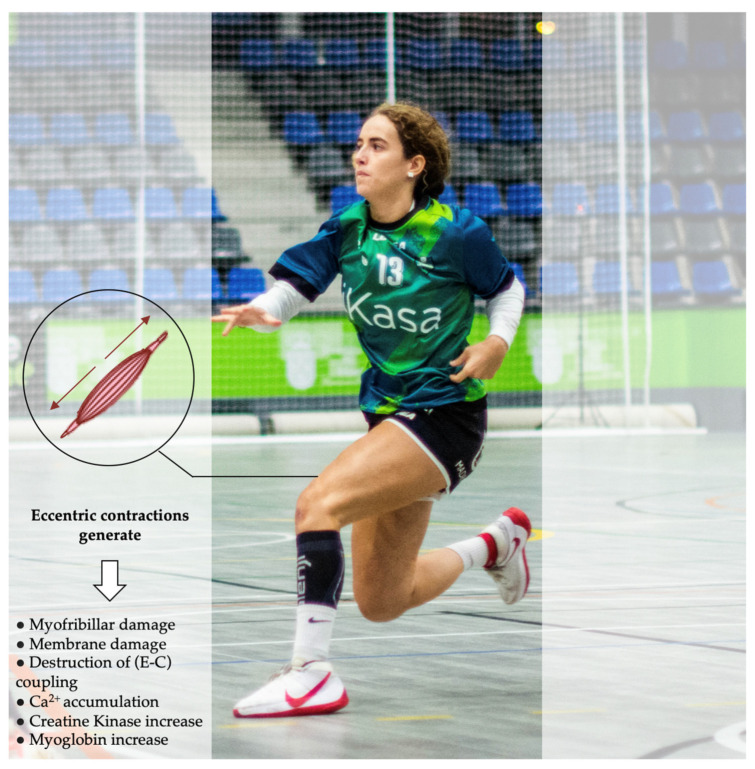
Physiological responses of repeated high-intensity decelerations.

Font et al. [15] indicated that wings, backs and pivots performed a similar number of accelerations and decelerations. On the other hand, HIA and HID were different between playing positions [15]. Wings (134.8 ± 60.7) performed slightly more HIA than backs (128.1 ± 55.2) and pivots (112.0 ± 33.6) (ES = 0.12 and 0.46, respectively); in contrast, backs (114.6 ± 51.77) performed a similar number of HID than wings (112.9 ± 56.0) and slightly more than pivots (99.6 ± 28.9) (ES = 0.03 and 0.3, respectively). These findings could suggest that wings performed more HIA because they have an important role in the counter-attack phase [45,46]; nevertheless, backs performed more HID because they have the main responsibility of building up the positional attack, which is characterised by a constant piston movement [62]. This handball-specific pattern is associated with intense eccentric contractions that generate important neuromuscular fatigue and tissue damage, especially if these high forces cannot be attenuated efficiently [63,64]; specifically, the physiological consequences of repeated HID (eccentric contractions) have been well documented (Figure 8) [65]. Therefore, strength and conditioning coaches should include different types of exercises during training sessions to develop the capacity of muscles and tendons to attenuate high eccentric forces, especially in back players [63,64]. Moreover, pivots performed fewer HIA and HID because their actions (e.g., high level of isometric force to block their opponent) do not always produce a movement or an acceleration [15,50].

As with accelerometery, PlayerLoad data were scarce. We found only four studies that provided information about PlayerLoad. All of the studies indicated that PlayerLoad was similar for wings, backs and pivots [13,15,41], except in the study carried out by Wik et al. [50]. However, PlayerLoad data should be interpreted and used with caution, because some researchers found that PlayerLoad calculations methods present many inconsistencies and lack clear and complete information [66]. Moreover, each trademark uses a different algorithm to calculate this variable (some manufacturers calculate PlayerLoad from three-dimensional accelerometery data, whereas others use two-dimensional data for the calculation) [20].

#### 4.1.5. Contextual Factors

The halves of the match had an impact on external load outcomes. Specifically, the time spent in high-intensity running during the match decreased in the second half [42,43,47]. Moreover, the total distance covered in the first 10 min was slightly higher than what was covered in the last 10 min of the game (ES = 0.33) [10]. In addition, initial values of PlayerLoad/min declined throughout halves [50].

In this line, it is well documented that exercise intensity decreases from the first to the second half of the match, suggesting that neuromuscular fatigue, muscle damage and inflammatory responses may occur during the game [10,42,43,47,50,67]. Moreover, repeated collisions and contacts may also further impair neuromuscular performance [63]. However, handball coaches can develop different player rotation strategies to avoid excessive physiological load and prevent fatigue during the match [10,50]. Moreover, strength and conditioning coaches should design training programmes focused on improving aerobic and anaerobic capacities with the aim of limiting a decrease in intensity after the most demanding periods of the games [50,68].

Finally, only two studies indicated that there were no differences between top-ranked and lower-ranked teams [10,12] or between winners and losers [12] in the total distance covered and running pace during the games. Therefore, more research is needed in this regard.

### 4.2. Internal Load

It is known that internal load can provide important information about energy systems’ contribution during the game. Accordingly, Povoas et al. [47] indicated that handball is an intermittent sport that primarily uses aerobic metabolism, interspersed with high-intensity actions that greatly tax anaerobic metabolism [69]. With the aim of knowing the contribution of the anaerobic glycolytic system, BLC data could be used, although this measure has several limitations (intensity of activity performed prior to the sampling, site and timing of the sampling and type of analyser) [3,70,71]. Despite these limitations, BLC represents a good starting point for understanding anaerobic glycolytic contributions during the game [3]. Thus, data from Michalsik et al. [44] and Povoas et al. [49] show that post-game BLCs were not greater than after the first half, demonstrating that the anaerobic glycolytic energy contribution was constant throughout the game. Furthermore, the anaerobic glycolytic contribution is playing-position-dependent because each position has different movement patterns and physical demands [3].

On the other side, the few existing studies on the contribution of aerobic system in handball were based on HR estimations [40,41,44,47,48,49]. Despite the fact that HR estimation has some limitations, it is the only way to collect information about aerobic demands because it is not possible to use a gas analyser to measure oxygen uptake during a game [3]. Considering this, our results indicated that the average HR ranged from 157.0 ± 18.0 to 185.3 ± 9.2 beats·min^−1^ during a game. Moreover, some studies [47,48,49] identified that handball players spend more than 50% of playing time >80% HRmax, suggesting that the aerobic system has an important contribution in handball.

Playing position and gender had an impact on the HR response. We found that goalkeepers have the lowest HR values during a game (from 70 ± 11.0% to 78.4 ± 5.9% Hrmax) compared with the other positions [9,48]. On the other hand, we found that pivots had the highest HR response (from 83.0 ± 9.0% to 90.1 ± 4.3% Hrmax) [40,48] and the lowest external load during the game. This fact indicates that external load is not enough for understanding the real physical demands in some playing positions (e.g., pivots and goalkeepers); therefore, we must combine it with internal load data. Finally, in regard to gender, some authors confirmed that female players have a higher %HR mean during the game compared with male players [40,48].

Despite the importance of the internal load data, we found only seven studies that provided information about that. For this reason, a reduced number of HR (*n* = 269) and BLC (*n* = 50) samples were included in this systematic review. Therefore, more research is needed in this regard.

### 4.3. Technical Activity

The technical activity was measured only in 5 of the 17 studies included in this systematic review, and all the studies focused on national competitions. Therefore, to date, no studies have analysed technical activity profiles in international competitions.

Considering the available data, the technical activity profile is different between playing positions and gender [45,46,47,48,49]. These different technical activity demands could induce different causes in injury patterns [72]. Therefore, this information is very important for designing optimal training programmes to maximise performance and for reducing injury risk.

Wings perform moderately more fast breaks than backs, so their training programmes should include more on-court sprinting, such as repeated sprint training (RST) or sprint interval training (SIT), and game-based intermittent endurance exercises, such as small-sided games (SSG) [73,74]. Moreover, they need to produce and maintain a high rate of force development (RFD) during the game for successful performance in accelerations and decelerations during transition phases (fast break and fast retreat) [68]. Finally, their training programmes should include exercises to prevent muscle injuries, specifically hamstring strain due to the high distance covered during high-speed running [3].

On the other hand, backs perform more throws than wings and pivots; therefore, coaches should develop injury prevention programmes to protect the shoulder against overuse injuries, specifically rotator cuff muscle and tendon injuries [3,75,76]. Moreover, backs perform more jumps, landings, stops and COD, so their training programmes should emphasise the prevention of knee injuries, especially in female players because they have an incidence of anterior cruciate ligament (ACL) injury that is 3–5 times higher than men [77,78]. Therefore, coaches should incorporate five types of interventions to prevent ACL injuries: (i) strength training (unilateral and bilateral exercises targeted on quadriceps, hamstrings, gluteus, the gastrocnemius and the soleus) [79,80,81,82]; (ii) landing and COD technique training (single- and double-leg landing stabilisation, pre-planned and unanticipated COD) with disturbances and opposition [79,80,81,83]; (iii) deceleration training (rapid decelerations from high speed with visual distractions) [84]; (iv) endurance training to minimising the appearance of fatigue [85]; and (v) core training [86].

Finally, pivots receive and give up more contacts (tackles and screenings). This fact suggests that they need more muscle hypertrophy and maximal strength than wings or backs [3,66]. More specifically, their training programmes should include exercises to increase the force of the core and the lower limb muscles with the aim of supporting heavy contacts and producing a high level of isometric force to block opponents [3,15].

In regard to gender, male players performed largely more fast breaks (6.9 ± 3.3) than female players (5.2 ± 2.8) [45,46]. In the same line, Þorgeirsson et al. [55] found a greater number of counterattack throws in men’s handball compared with women’s handball. This fact could indicate that male players need more endurance training to repeat high-intensity actions during the entire match; however, this information should be interpreted with caution because the running pace was largely greater in female competitions (110.5 ± 7.2 m∙min^−1^) [40] compared with male competitions (78.4 ± 19.7 m∙min^−1^) [10,11,12,15].

### 4.4. Limitations

This systematic review has some limitations: (i) The final sample was composed of a small number of female players (*n* = 133). (ii) A reduced number of HR and BLC samples were collected. (iii) The external load was analysed with different methodologies and technology (TMA or LPS). (iv) A lack of clarification and consensus across different researchers and manufacturers´ tracking systems on which the standardise speed ranges or thresholds for locomotive categories (standing still, walking, jogging, running, high-intensity running and sprinting) are based upon may make it difficult to generalise the findings to other populations. (v) PlayerLoad data differed depending on the device used, because each trademark uses a different algorithm to calculate this variable. Moreover, PlayerLoad calculation methods present many inconsistencies and a lack of clear and complete information. This fact represents a disadvantage for comparing and generalising the scarce existing data in handball. (vi) The is a lack of information about the management of the players´ rotation by coaches, which is directly related to the physical load of the player. (vii) The physical demands analysis is based mainly on player displacements, but some technical actions, such as blocks or screenings, represent a high load and do not produce a movement or an acceleration. Therefore, some tracking systems do not allow registering the load produced by these types of actions.

### 4.5. Practical Applications

This study is a starting point for further research into the physical demands of elite handball. Some of the possible practical applications in this regard are as follows: (i) individualise training programmes according to playing position, gender and competitive level by handball coaches and strength and conditioning professionals; (ii) improve the players’ strength (maximal and explosive-type RFD) and endurance to repeat high-intensity actions during the entire match; (iii) design and implement injury prevention programmes according to the technical activity profile of each playing position (e.g., backs should develop the capacity of muscles and tendons to attenuate high eccentric forces associated with intense decelerations, pivots should increase the force of the core with the aim of supporting heavy contacts and wings should improve the force of the lower limb muscle to prevent hamstring strains due to the high distance covered at high-speed running); (iv) incorporate different type of interventions to prevent ACL injuries (especially in female players): strength training, landing and COD technique training with disturbances and opposition, deceleration training and endurance training to minimise the appearance of fatigue and core training; (v) develop different players´ rotation strategies to avoid excessive physiological loading and prevent fatigue during the match; and (vi) incorporate tracking systems to control and prescribe physical load during training practice and competition.

### 4.6. Future Lines of Research

Further studies will be needed to deepen our knowledge in the following topics: (i) physical demands in elite female handball; (ii) physical demands of the goalkeeper; (iii) physical demands during the most demanding scenarios or passages of handball games, because the average values could underestimate the real physical demands; (iv) relationship between technical–tactical situations and physical demands; (v) effects of contextual factors (halves of the match, match outcome, score differential or quality of opposition) on external load; and (vi) create physical profiles according to playing positions, gender and competition level to improve talent identification and development systems. Finally, it would be helpful to standardise LPS in order to have a valid and reliable technology to track players’ performance.

## 5. Conclusions

This systematic review confirms the differences in relation to physical demands depending on playing positions, competition level and gender. First, competition level affected the total distance covered; specifically, we found that the total distance covered was largely greater in national competitions compared with international competitions; however, the running pace did not present any significant difference between the international and national levels. Regarding gender, the total distance covered was moderately greater in female competitions compared with male competitions, and the running pace was largely greater in female competitions compared with male competitions. In relation to playing position, backs and wings covered a moderately greater total distance and slightly more meters per minute than pivots. Therefore, this research provides practical applications for handball coaches and strength and conditioning professionals in designing and implementing more individualised training programmes to maximise performance and reduce injury risks.

## Figures and Tables

**Table 1:** Summary of the sample characteristics and methods.

	Sample Characteristics									Methods	
Authors	N	Playing Position	Competitive Level	Gender	Age (Years)	Body Mass (kg)	Height (cm)	Body Fat (%)	Experience	Data Records	Technology
Belka et al. [40]	14	Wings (*n* = 5)	3rd Level (Czech Republic’s elite juniors’ handball league)	Female	17.8 ± 0.4	62.4 ± 3.0	169.4 ± 6.7	-	10 years of experience in the sport	6 matches (3 home and 3 away)	HR and TMA
		Pivots (*n* = 2)			18.0 ± 0.0	68.0 ± 3.0	166.0 ± 4.0				
		Backs (*n* = 7)			17.9 ± 0.3	66.7 ± 8.7	170.9 ± 7.3				
Cardinale et al. [10]	384	Undefined	1st Level (24 National Teams from WCH 2015)	Male	27.9 ± 4.4	67.8 ± 35.2	191 ± 10.0	-	-	2505 records in 88 matches	TMA
Font et al. [15]	16	Wings (*n* = 5)	2nd Level (First Division Spain)	Male	26.6 ± 6.3	83.2 ± 4.1	183.1 ± 4.4	-	-	188 records in 14 official home matches	LPS (Wimu^TM^)
		Pivots (*n* = 3)			28.3 ± 4.0	198.0 ± 8.4	101.5 ± 4.9				
		Backs (*n* = 8)			29.1 ± 5.9	194.1 ± 1.9	95.8 ± 5.0				
Kniubaite et al. [41]	8	Undefined	2nd Level (LMRL and WBHL)	Female	23.0 ± 2.1	67.8 ± 6.8	173.5 ± 4.9	20.4 ± 3.9	-	112 records in 14 matches (7 LMRL and 7 WBHL)	HR and LPS (Catapult^TM^)
Luteberget et al. [13]	20	Undefined	1st Level (Golden League Tournament)	Female	25 ± 3.8	-	175.3 ± 4.5	-	-	97 records in 9 matches	LPS (Catapult^TM^)
Manchado et al. [9]	25	Wings (*n* = 8)	1st Level (Norway National Team) and 2nd Level (First Division Germany)	Female	25.2 ± 3.2	67.8 ± 4.8	175.2 ± 6.3	-	-	2 matches (1 match of an international tournament and 1 match of German First League)	HR and TMA
		Pivots (*n* = 5)									
		Backs (*n* = 9)									
		Goalkeepers (*n* = 3)									
Manchado et al. [11]	40	Undefined	1st Level (4 Teams from EHF Champions League 2019/2020)	Male	29.7 ± 4.9	91.2 ± 12.5	191.1 ± 8.6	-	-	133 records in 4 matches	LPS (Kinexon^TM^)
Manchado et al. [12]	414	Left wing (*n* = 48)	1st Level (24 National Teams from EURO 2020)	Male	28.3 ± 4.6	84.4 ± 7.9	186.9 ± 5.7	-	-	1865 records in 71 matches	LPS (Kinexon^TM^)
		Left back (*n* = 73)			26.8 ± 4.7	97.2 ± 6.5	196.1 ± 4.2				
		Center back (*n* = 55)			27.5 ± 5.0	97.2 ± 6.5	189.7 ± 5.8				
		Right back (*n* = 52)			27.9 ± 4.8	95.7 ± 8.9	194.4 ± 5.8				
		Right wing (*n* = 50)			28.0 ± 4.4	83.1 ± 6.3	184.6 ± 5.4				
		Pivots (*n* = 79)			28.5 ± 4.7	105.3 ± 8.5	196.8 ± 4.6				
Michalsik et al. [42]	26	Wings (*n* = 9)	1st Level (24 National Teams from EURO 2020)	Male	24.9 ± 2.6	185.8 ± 5.3	80.9 ± 5.5	-	7.2 ± 3.6 years of playing experience at senior elite level	82 records in 62 matches	TMA
		Pivots (*n* = 7)			27.7 ± 2.3	194.7 ± 2.1	101.4 ± 8.3				
		Backs (*n* = 7)			26.2 ± 3.4	187.0 ± 6.4	91.7 ± 6.7				
		Goalkeepers (*n* = 3)			26.8 ± 2.4	188.7 ± 5.5	94.3 ± 6.8				
Michalsik et al. [43]	24	Wings (*n* = 10)	2nd Level (First Division Denmark)	Female	25.4 ± 4.6	65.2 ± 2.7	170.6 ± 5.0	-	6.9 ± 3.3 years of playing experience at adult elite level	180 single-player recordings in 46 matches	TMA
		Pivots (*n* = 7)			26.3 ± 3.2	76.5±8.1	178.8 ± 3.4				
		Backs (*n* = 7)			26.2 ± 3.8	71.4 ± 6.1	175.1 ± 5.3				
Michalsik et al. [44]	26	Wings (*n* = 9)	2nd Level (First Division Denmark)	Male	24.9 ± 2.6	185.8 ± 5.3	80.9 ± 5.5	-	7.2 ± 3.6 years of playing experience at senior elite level	41 HR samples and 38 BLC samples	HR and BLC
		Pivots (*n* = 7)			27.7 ± 2.3	194.7 ± 2.1	101.4 ± 8.3				
		Backs (*n* = 7)			26.2 ± 3.4	187.0 ± 6.4	91.7 ± 6.7				
		Goalkeepers (*n* = 3)			26.8 ± 2.4	188.7 ± 5.5	94.3 ± 6.8				
Michalsik et al. [45]	24	Wings (*n* = 10)	2nd Level (First Division Denmark)	Female	25.4 ± 4.6	65.2 ± 2.7	170.6 ± 5.0	-	6.9 ± 3.3 years of playing experience at adult elite level	180 single-player recordings in 46 tournament matches	TMA
		Pivots (*n* = 7)			26.3 ± 3.2	76.5±8.1	178.8 ± 3.4				
		Backs (*n* = 7)			26.2 ± 3.8	71.4 ± 6.1	175.1 ± 5.3				
Michalsik et al. [46]	26	Wings (*n* = 9)	2nd Level (First Division Denmark)	Male	24.9 ± 2.6	185.8 ± 5.3	80.9 ± 5.5	-	7.2 ± 3.6 years of playing experience at senior elite level	82 records in 62 tournament matches	TMA
		Pivots (*n* = 7)			27.7 ± 2.3	194.7 ± 2.1	101.4 ± 8.3				
		Backs (*n* = 7)			26.2 ± 3.4	187.0 ± 6.4	91.7 ± 6.7				
		Goalkeepers (*n* = 3)			26.8 ± 2.4	188.7 ± 5.5	94.3 ± 6.8				
Povoas et al. [47]	30	Wings (*n* = 10)	2nd Level (First Division Portugal)	Male	25.2 ± 3.6	87.7 ± 9.0	186.5 ± 7.9	-	At least 5 years of experience in the First Portuguese League	60 HR samples and 60 TMA records in 10 matches	HR and TMA
		Pivots (*n* = 10)									
		Backs (*n* = 10)									
Povoas et al. [48]	40	Wings (*n* = 10)	2nd Level (First Division Portugal)	Male	24.6 ± 2.8	80.5 ± 6.1	177.3 ± 5.0	10.5 ± 3.2	At least 5 years of experience in the top Portuguese handball professional league	70 HR samples and 70 TMA records in 10 matches	HR and TMA
		Pivots (*n* = 10)			24.4 ± 3.9	98.6 ± 4.9	192.0 ± 2.7	10.0 ± 2.4			
		Backs (*n* = 10)			25.7 ± 4.1	89.8 ± 7.4	191.0 ± 5.6	8.9 ± 1.5			
		Goalkeepers (*n* = 10)			26.2 ± 4.1	87.4 ± 8.7	189.8 ± 2.2	10.0 ± 0.8			
Povoas et al. [49]	40	Wings (*n* = 13)	2nd Level (First Division Portugal)	Male							
		Pivots (*n* = 14)									
		Backs (*n* = 13)									
Wik et al. [50]	18	Undefined	1st Level (Golden League International Tournament)	Female	25.1 ± 3.8	-	-	-	-	85 records in 9 matches	LPS (Catapult^TM^)
					24.3 ± 2.9						
					26.0 ± 4.1						

Notes: Undefined = no specified playing position; LMRL = First Division Lithuanian Women’s League (Lietuvos Moteru Rankinio Lyga); WBHL = Women’s Baltic Handball League; HR = heart rate; TMA = time–motion analysis; LPS = local positioning system; BLC = blood lactate concentrations.

**Table 2:** External load variables based on the local positioning system.

External Load		Author								
		Font et al. [15]						Kniubaite et al. [41]		
		Wings (*n* = 5)		Pivots (*n* = 3)		Backs (*n* = 8)		Undefined		
DISTANCE	Playing time (min:s)	60.8 ± 6.9		56.3 ± 12.0		60.7 ± 12.5		-		
	Total distance covered (m)	3903.0 ± 1224.0		3149 ± 630.0		3688 ± 899.0		-		
	Running pace (m∙min^−1^)	64.5 ± 10.4		56.5 ± 6.6		61.9 ± 8.8		-		
	High-intensity running (m)	410.3 ± 193.2		172.4 ± 96.0		182.1 ± 115.1		-		
	Sprinting (m)	98.0 ± 75.4		40.0 ± 30.0		42.6 ± 52.9		-		
ACCEL	HIA/game (n)	134.8 ± 60.7		112.0 ± 33.6		121.1 ± 55.2		-		
	HID/game (n)	112.9 ± 56.0		99.6 ± 28.9		114.6 ± 51.7		-		
	HIE∙min (n∙min^−1^)	-		-		-		-		
LOAD	PlayerLoad (u.a.)	68.1 ± 23.1		59.5 ± 12.0		64.5 ± 16.4		335.0 ± 142.3		
	PlayerLoad∙min (u.a.∙min^−1^)	1.1 ± 0.2		1.1 ± 0.2		1.1 ± 0.2		9.3 ± 2.1		
Notes: ACCEL (accelerometery); Undefined = no specified playing position; high-intensity running: distance covered from 5.9 to 6.7 m∙s^−1^; sprinting: distance covered faster than 6.7 m∙s^−1^; HIA/game (high-intensity accelerations) = total number of accelerations greater than 2.0 g during a game; HID/game (high-intensity decelerations) = total number of decelerations greater than 2.0 g during a game; HIE∙min (high-intensity events per minute) = it is calculated from the sum of the accelerations, decelerations and changes of direction greater than 2.5 m∙s^−2^; U.A. (arbitrary units).										
**External Load**		**Author**								
		**Luteberget et al.** [13]						**Manchado et al.** [11]		
		**Wings** **(*n* = 25** **Records)**		**Pivots** **(*n* = 14** **Records)**	**Backs** **(*n* = 44** **Records)**	**Goalkeepers** **(*n* = 14** **Records)**		**Offensive Players** **(*n* = 66 Records)**		**Defensive Players** **(*n* = 67 Records)**
DISTANCE	Playing time (min:s)	31.4 ± 14.7		34.4 ± 12.5	30.9 ± 16.0	42.2 ± 16.6		15.6 ± 8.0		15.4 ± 8.9
	Total distance covered (m)	-		-	-	-		1388.2 ± 2627.1		1305.4 ± 5059.6
	Running pace (m∙min^−1^)	-		-	-	-		88.4 ± 20.7		80.8 ± 27.1
	High-intensity running (m)	-		-	-	-		-		-
	Sprinting (m)	-		-	-	-		-		-
ACCEL	HIA/game (n)	-		-	-	-		-		-
	HID/game (n)	-		-	-	-		-		-
	HIE∙min (n∙min^−1^)	3.9 ± 1.5						-		-
LOAD	PlayerLoad (u.a.)	-		-	-	-		-		-
	PlayerLoad∙min (u.a.∙min^−1^)	8.8 ± 2.1						-		-
Notes: ACCEL (accelerometery); HIA/game (high-intensity accelerations) = total number of accelerations greater than 2.0 g during a game; HID/game (high-intensity decelerations) = total number of decelerations greater than 2.0 g during a game; HIE∙min (high-intensity events per minute) = it is calculated from the sum of the accelerations, decelerations and changes of direction greater than 2.5 m∙s^−2^; U.A. (arbitrary units).										
**External Load**		**Author**								
		**Manchado et al.** [12]						**Wik et al.** [50]		
		**Left Wing** **(*n* = 213 Records)**	**Left Back** **(*n* = 320 Records)**	**Center Back** **(*n* = 248 Records)**	**Right Back** **(*n* = 246 Records)**	**Right Wing** **(*n* = 230 Records)**	**Pivots** **(*n* = 374 Records)**	**Wings** **(*n* = 24 Records)**	**Pivots** **(*n* = 15 Records)**	**Backs** **(*n* = 46 Records)**
DISTANCE	Playing time (min:s)	32.0 ± 17.0	23.7 ± 12.5	24.9 ± 13.6	24.4 ± 13.3	29.9 ± 18.4	24.5 ± 13.8	29.5 ± 14.1	28.7 ± 15.0	30.9 ± 14.6
	Total distance covered (m)	2547.1 ± 1309.5	1887.9 ± 962.2	2194.3 ± 1093.9	1943.2 ± 1003.0	2371.8 ± 1456.8	1835.2 ± 979.1	-	-	-
	Running pace (m∙min^−1^)	83.6 ± 23.5	90.8 ± 35.5	98.3 ± 36.1	86.5 ± 30.1	85.1 ± 32.9	91.2 ± 42.7	-	-	-
	High-intensity running (m)	337.9 ± 202.4	73.8 ± 73.7	103.9 ± 74.4	70.8 ± 50.9	320.5 ± 221.4	92.1 ± 62.9	-	-	-
	Sprinting (m)	52.2 ± 46.5	4.3 ± 10.7	5.4 ± 9.4	2.9 ± 6.3	40.7 ± 40.5	1.5 ± 4.7	-	-	-
ACCEL	HIA/game (n)	-	-	-	-	-	-	-	-	-
	HID/game (n)	-	-	-	-	-	-	-	-	-
	HIE∙min (n∙min^−1^)	-	-	-	-	-	-	-	-	
LOAD	PlayerLoad (u.a.)	-	-	-	-	-	-	-	-	-
	PlayerLoad∙min (u.a.∙min^−1^)	-	-	-	-	-	-	9.1 ± 0.6	9.7 ± 1.4	9.3 ± 0.8
Notes: ACCEL (accelerometery); high-intensity running: distance covered from 5.5 to 6.9 m∙s^−1^; sprinting: distance covered faster than 7.0 m∙s^−1^; HIA/game (high-intensity accelerations) = total number of accelerations greater than 2.0 g during a game; HID/game (high-intensity decelerations) = total number of decelerations greater than 2.0 g during a game; HIE∙min (high-intensity events per minute) = it is calculated from the sum of the accelerations, decelerations and changes of direction greater than 2.5 m∙s^−2^; U.A. (arbitrary units).										

**Table 3:** External load variables based on time–motion analysis.

Locomotive Categories		Authors						
		Belka et al. [40]	Cardinale et al. [10]	Manchado et al. [9]	Michalsik et al. [42]	Michalsik et al. [43]	Povoas et al. [47]	Povoas et al. [48]
STANDING STILL	FT (%)	-	1.1 ± NA	-	36.8 ± NA	10.8 ± NA	43 ± 9.2	43 ± 6.9
	TDC (m)	0	-	-	0	0	0	0
WALKING	FT (%)	-	50.4 ± NA	30.8 ± 5.9	39.6 ± NA	62.3 ± NA	35.0 ± 6.9	34.9 ± 5.1
	TDC (m)	444.4 ± 218.1	-	961 ± 539.0	1424 ± 265.0	2103 ± 334.0	2002 ± 427.2	2001 ± 313.0
JOGGING	FT (%)	-	26.6 ± NA	-	8.6 ± NA	18.8 ± NA	8.8 ± 3.1	8.7 ± 2.4
	TDC (m)	1777 ± 268.1	-	-	618 ± 155.0	1114 ± 219.0	1014 ± 334.8	1014 ± 252.6
RUNNING	FT (%)	-	14.8 ± NA	29.1 ± 3.8	4.4 ± NA	4.9 ± NA	-	-
	TDC (m)	1761.1 ± 269.7	-	761.0 ± 420.0	510 ± 121.0	496 ± 252.0	-	-
HIGH-INTENSITY RUNNING	FT (%)	-	6.1 ± NA	29.7 ± 3.9	1.4 ± NA	0.7 ± NA	2.2 ± 1.2	2.1 ± 1.0
	TDC (m)	1223.7 ± 223.6	-	752.0 ± 484.0	207 ± 91.0	93 ± 67.0	508 ± 281.6	508 ± 245.6
SPRINTING	FT (%)	-	1 ± NA	10.5 ± 4.1	0.4 ± NA	0.2 ± NA	0.4 ± 0.3	0.3 ± 0.2
	TDC (m)	1589.9 ± 233.4	-	272.0 ± 224.0	78 ± 91.0	10 ± 11.0	107 ± 87.3	107 ± 73.3

Notes: FT = Fraction of total time; TDC = total distance covered; NA = not available; Belka et al. [40] = walking from 0.4 to 3.0 km/h, jogging from 3.1 to 8.0 km/h, running from 8.1 to 13.0 km/h, high-intensity running from 13.1 to 18 km/h, sprinting faster than 18.1 km/h; Cardinale et al. [10] = walking from 0.2 to 1.9 m∙s^−1^, jogging from 2.0 to 3.9 m∙s^−1^, running from 4.0 to 5.4 m∙s^−1^, high-intensity running from 5.5 to 6.9 m∙s^−1^, sprinting faster than 7.0 m∙s^−1^; Manchado et al. [9] = walking from 0.1 to 1.3 m∙s^−1^, running from 1.4 to 3.0 m∙s^−1^, high-intensity running from 3.1 to 5.2 m∙s^−1^, sprinting faster than 5.2 m∙s^−1^; Michalsik et al. [42,43] = walking from 4.0 to 7.9 km/h, jogging from 8.0 to 12.9 km/h, running from 13.0 to 16.9 km/h, high-intensity running from 17.0 to 23.9 km/h, sprinting faster than 24.0 km/h; Povoas et al. [47,48] = walking from 0.37 to 3.6 km/h, jogging from 3.7 to 10.8 km/h, high-intensity running from 18.1 to 25.0 km/h, sprinting faster than 25.1 km/h.

**Table 4:** Internal load variables.

Author	Sample Characteristics	Internal Load				
		Heart Rate Mean		Heart Rate Peak		Blood Lactate
	Playing Position	%HRmax	Beats·min^–1^	%HRmax	Beats·min^–1^	mmol·L^−1^
Belka et al. [40]	Wings (*n* = 5)	89.9 ± 3.5	183.8 ± 6.2	-	-	-
	Pivots (*n* = 2)	90.1 ± 4.3	185.3 ± 9.2			
	Backs (*n* = 7)	89.2 ± 4.4	182.9 ± 8.4			
Kniubaite et al. [41]	Undefined	84.8 ± 5.1	-	-	-	-
Manchado et al. [9]	Wings (*n* = 8)	86.5 ± 4.5	-	-	195 ± 1.0	-
	Pivots (*n* = 5)					
	Backs (*n* = 9)					
	Goalkeepers (*n* = 3)	78.4 ± 5.9				
Michalsik et al. [44]	Wings (*n* = 9)	-	163 ± 6.0	-	-	Pre-match: 1.5 ± 0.5 After 1st half: 3.7 ± 1.6 After 2nd half: 4.8 ± 1.9 Post-match: 2.8 to 10.8
	Pivots (*n* = 7)					
	Backs (*n* = 7)					
	Goalkeepers (*n* = 3)					
Povoas et al. [47]	Wings (*n* = 10)	-	157 ± 18.0	-	185 ± 9.6	-
	Pivots (*n* = 10)					
	Backs (*n* = 10)					
Povoas et al. [48]	Wings (*n* = 10)	79 ± 10.0	-	95 ± 4.0	-	-
	Pivots (*n* = 10)	83 ± 9.0	-	98 ± 2.0	-	
	Backs (*n* = 10)	84 ± 9.0	-	96 ± 4.0	-	
	Goalkeepers (*n* = 10)	70 ± 11.0	-	90 ± 7.0	-	
Povoas et al. [49]	Wings (*n* = 13) Pivots (*n* = 14) Backs (*n* = 13)	83 ± 8.0	159 ± 17.0	96 ± 4.0	187 ± 9.0	After 1st half: 4.2 ± 2.3 After 2nd half: 3.1 ± 1.8 Mean: 3.6 ± 2.1 Peak: 8.0 ± 1.4

Notes: Undefined = no specified playing position; %HRmax = percentage of heart rate maximum.

**Table 5:** The study quality analysis (‘STROBE’ checklist).

Reference	Title and Abstract	Introduction		Methods									Results					Discussion				Other Information	Strobe Points
	1	2	3	4	5	6	7	8	9	10	11	12	13	14	15	16	17	18	19	20	21	22	
Belka et al. [40]	1	1	1	0	1	1	1	1	0	1	1	1	1	1	1	1	0	1	0	1	1	1	18
Cardinale et al. [10]	1	1	1	0	1	1	1	1	0	1	1	1	1	1	1	1	1	1	1	1	1	1	20
Font et al. [15]	1	1	1	1	1	1	1	1	0	1	1	1	1	1	1	1	1	1	1	1	1	1	21
Kniubaite et al. [41]	1	1	1	1	1	1	1	1	0	1	1	1	1	1	1	1	1	1	1	1	1	0	21
Luteberget et al. [13]	1	1	1	0	1	1	1	1	0	0	1	1	1	0	1	1	0	1	1	1	1	0	17
Manchado et al. [9]	1	1	1	1	1	1	1	1	0	0	1	1	1	1	1	1	1	1	0	1	1	0	18
Manchado et al. [11]	1	1	1	1	1	1	1	1	0	1	1	1	1	1	1	1	1	1	1	1	1	1	21
Manchado et al. [12]	1	1	1	1	1	1	1	1	0	1	1	1	1	1	1	1	1	1	1	1	1	1	21
Michalsik et al. [42]	1	1	1	0	1	1	1	1	0	1	1	1	1	1	1	1	0	1	0	1	0	0	16
Michalsik et al. [43]	1	1	1	0	1	1	1	1	0	1	1	1	1	1	1	1	0	1	0	1	0	0	16
Michalsik et al. [44]	1	1	1	0	1	1	1	1	0	1	1	1	1	1	1	1	0	1	0	1	1	0	17
Michalsik et al. [45]	1	1	1	1	1	1	1	1	0	1	1	1	1	1	1	1	0	1	0	1	1	1	19
Michalsik et al. [46]	1	1	1	1	1	1	1	1	0	1	1	1	1	1	1	1	0	1	0	1	1	1	19
Povoas et al. [47]	1	1	1	1	1	1	1	1	0	0	1	1	1	1	1	1	0	1	1	1	0	1	18
Povoas et al. [48]	1	1	1	1	1	1	1	1	0	0	1	1	1	1	1	1	0	1	1	1	0	1	18
Povoas et al. [49]	1	1	1	1	1	1	1	1	0	0	1	1	1	1	1	1	0	1	1	1	0	1	18
Wik et al. [50]	1	1	1	1	1	1	1	1	0	0	1	1	1	0	1	1	1	1	1	1	1	0	18

Notes: ‘0′ = item with absence or lack of information; ‘1′ = item with complete and explicit information; in Title and Abstract, 1 (Title/Abstract) = include information on the sport, athlete population (sex, age, geographic region), level of competition, and the duration of observation. In Introduction, 2 (Background) = explain the scientific background and rationale for the investigation being reported. 3 (Objectives) = state specific objectives, including any prespecified hypotheses. In Methods, 4 (Study Design) = present key elements of study design early in the paper; 5 (Settings) = describe the setting, locations, and relevant dates, including periods of recruitment, exposure, follow-up, and data collection; 6 (Participants) = define the population of athletes as well as describe how they were selected and recruited; 7 (Variables) = clearly define all outcomes, exposures, predictors, potential confounders and effect modifiers. Give diagnostic criteria, if applicable; 8 (Data Sources) = for each variable of interest, provide sources of data and details of methods of assessment (measurement). Describe comparability of assessment methods if there is more than one group; 9 (Bias) = describe any efforts that address potential sources of bias; 10 (Study Size) = explain how the study size was arrived at; 11 (Quantitative Variables) = explain how quantitative variables were handled in the analyses. If applicable, describe which groupings were chosen and why; 12 (Statistical Methods) = describe all the statistical methods, including those used to control for confounding/describe any methods used to examine subgroups and interactions/explain how missing data were addressed/describe any sensitivity analyses. In Results, 13 (Participants) = report numbers of individuals at each stage of study/provide the reasons for non-participation at each stage; 14 (Descriptive Data) = give characteristics of study participants (e.g., demographic, clinical and social) and information on exposures and potential confounders; 15 (Outcome Data) = report numbers of outcome events or summary measures over time; 16 (Main Results) = give unadjusted estimates and, if applicable, confounder-adjusted estimates and their precision (e.g., 95% confidence interval). Make clear which confounders were adjusted for and why they were included; 17 (Other Analyses) = report other analyses conducted—e.g., analyses of subgroups and interactions and sensitivity analyses. In Discussion, 18 (Key Results) = summarize key results with reference to study objectives; 19 (Limitations) = discuss limitations of the study, taking into account sources of potential bias or imprecision. Discuss both the direction and magnitude of any potential bias; 20 (Interpretation) = give a cautious overall interpretation of results considering objectives, limitations, multiplicity of analyses, results from similar studies and other relevant evidence; 21 (Generalizability) = discuss the generalisability (external validity) of the study results. In Other Information, 22 (Funding) = give the source of funding and the role of the funders for the present study and, if applicable, for the original study on which the present article is based.
